# Identification of the Immune Cell Infiltration Landscape in Hepatocellular Carcinoma to Predict Prognosis and Guide Immunotherapy

**DOI:** 10.3389/fgene.2021.777931

**Published:** 2021-11-25

**Authors:** Shiyan Yang, Yajun Cheng, Xiaolong Wang, Ping Wei, Hui Wang, Shanzhong Tan

**Affiliations:** ^1^ Department of Integrated TCM and Western Medicine, Nanjing Hospital Affiliated to Nanjing University of Chinese Medicine, Nanjing, China; ^2^ Department of Gastroenterology, Huaian Hospital Affiliated to Xuzhou Medical University, Huaian, China; ^3^ Department of Gastroenterology, People’s Hospital of Lianshui, Huaian, China; ^4^ The Department of General Surgery, Tumor Hospital of Huaian, Huaian, China; ^5^ The Department of Ultrasound, Huaian Hospital Affiliated to Xuzhou Medical University, Huaian, China; ^6^ The Department of Rehabilitation Medicine, Huaian Hospital Affiliated to Xuzhou Medical University, Huaian, China

**Keywords:** carcinoma, immune cell infiltration landscape, immunotherapy, ICI scores, prognosis

## Abstract

**Background:** Globally, hepatocellular carcinoma (HCC) is the sixth most frequent malignancy with a high incidence and a poor prognosis. Immune cell infiltration (ICI) underlies both the carcinogenesis and immunogenicity of tumors. However, a comprehensive classification system based on the immune features for HCC remains unknown.

**Methods:** The HCC dataset from The Cancer Genome Atlas (TCGA) and International Cancer Genome Consortium (ICGC) cohorts was used in this study. The ICI patterns of 571 patients were characterized using two algorithms: the patterns were determined based on the ICI using the ConsensusClusterPlus package, and principal component analysis (PCA) established the ICI scores. Differences in the immune landscape, biological function, and somatic mutations across ICI scores were evaluated and compared, followed by a predictive efficacy evaluation of ICI scores for immunotherapy by the two algorithms and validation using an external immunotherapy cohort.

**Results:** Based on the ICI profile of the HCC patients, three ICI patterns were identified, including three subtypes having different immunological features. Individual ICI scores were determined; the high ICI score subtype was characterized by enhanced activation of immune-related signaling pathways and a significantly high tumor mutation burden (TMB); concomitantly, diminished immunocompetence and enrichment of pathways associated with cell cycle and RNA degradation were found in the low ICI score subtype. Taken together, our results contribute to a better understanding of an active tumor and plausible reasons for its poor prognosis.

**Conclusion:** The present study reveals that ICI scores may serve as valid prognostic biomarkers for immunotherapy in HCC.

## Introduction

HCC is an aggressive malignancy that frequently develops and progresses in the setting of chronic liver disease or cirrhosis (Park et al., 2015). Statistics from 2018 indicate that HCC is the sixth most frequently occurring malignancy and the fourth highest cause of cancer-related deaths (Kulik and El-Serag, 2019). To date, approximately 841,000 new cases are registered and more than 782,000 HCC-related deaths are recorded (Singal et al., 2020). Alcohol consumption, obesity, fatty liver, and hepatitis infection are some of the important risk factors for HCC (Caruso et al., 2021). Current advances in HCC-diagnosis, surgical treatment, transplantation, chemotherapy, radiotherapy, and targeted molecular therapies, to some extent, have improved the prognosis of HCC patients (Fan, 2012), but the majority of the diagnosed patients are already at an advanced stage and have only limited conservative treatment options. The rate of cure in HCC remains low due to its high malignancy, recurrence rate, increased metastasis, and adverse response to chemotherapy (Pillai et al., 2020; Farzaneh et al., 2021).

As a treatment for HCC, despite its limited efficacy, immunotherapy has yielded promising results (Silva et al., 2020). However, the benefits of immunotherapy are largely limited to only a small number of HCC patients. Existing studies have shown that immune-associated genes and lymphocytes infiltrating tumors play a key role in tumor oncogenesis and its progression (Wang et al., 2020); the dynamic interactions between immune cells infiltration into the tumor microenvironment, cytokines secreted by immune cell types, and cancerous cells are involved in HCC tumor progression (Choi and Park, 2017; Sachdeva and Arora, 2020). A clearer understanding of these specific dynamical patterns may be beneficial for immunotherapy. Therefore, detailed investigations of the immune landscape of the tumor microenvironment (TME) and identification of ideal HCC subgroups for immunotherapy are important to improve the immunotherapeutic responses and prognostic prediction (Hosseinzadeh et al., 2018; Robert et al., 2020).

Extensive studies on the TME indicate the critical functions of infiltrating immune cells in tumor dissemination, recurrence, metastatic activity, and immunotherapeutic responses (Jiang et al., 2018a; Zeng et al., 2018). As an example, CD8^+^ T cells are potent regulators of adaptive immunity as they can eliminate pathogen-infected and tumor cells (Stairiker et al., 2020), and thus, critically affect tumor immunity (Han et al., 2020). Tumor-associated macrophages (TAM) exert multiple tumor-beneficial effects through the secretion of immunosuppressive cytokines, associated with unfavorable prognoses (De Palma and Lewis, 2013; Noy and Pollard, 2014). Through their inhibitory activity, M2-type macrophages critically regulate the tumor microenvironment (Mehla and Singh, 2019). Taken together, these studies suggest that immune cell interactions in TME may provide new insights for cancer therapy. However, a comprehensive and clear understanding of immune landscape complexity in HCC is still lacking.

Here, we evaluated the immune landscape of HCC using the CIBERSOFT algorithm. Based on their ICI features, the HCC patients were classified into four subtypes. Subsequently, based on immune subtypes, ICI scores were established to further assess the immune landscape of HCC, for accurate prognostic prediction of the patients and their immunotherapeutic responses.

## Materials and Methods

### Hepatocellular Carcinoma Sample and Data Acquisition

Patients with complete clinical information (Stage, Follow-up Information, Age and Gender) were selected in this study, after removing patients who did not meet the criteria. RNA-Seq data of 340 HCC patients and their complete corresponding clinical information were acquired from The Cancer Genome Atlas (TCGA) using the GDC API; for the training cohort, expression data in FPKM (Fragments Per Kilobase per Million) were subsequently TPM-normalized (Transcripts Per Kilobase per Million). In addition, RNA-Seq data of 231 HCC patients and their complete corresponding clinical information were obtained from the International Cancer Genome Consortium (ICGC) database (Fujimoto et al., 2016). Similarly, for the validation set, the raw sequencing data were TPM normalized.

### Evaluation of Immune Infiltration Levels and Consensus Clustering

The level of infiltration of the 22 immune cells was quantified for each sample of the HCC-TCGA cohort using the “CIBERSORT” R package with the LM22 signature (Newman et al., 2015). Next, the ESTIMATE algorithm was used to compute the scores for immune and stromal characteristics for each patient (Yoshihara et al., 2013). Hierarchical consensus clustering for HCC was performed for each sample based on the individual pattern of ICI. In this analysis, the PAM unsupervised clustering based on Pearson’s correlation and Ward’s linkage based on the “ConsensusClusterPlus” R package, were used (Yu et al., 2012) and repeated 1,000 times to reduce sampling errors and ensure a stable classification. Consensus clustering is a popular bioinformatics algorithm, which was extensively utilized in cancer-related studies (Liu et al., 2021a; Liu et al., 2021b; Liu et al., 2021c; Liu et al., 2021d).

### DEGs Identified Based on ICI Phenotype

Patients were subdivided based on ICI and were referred to as the ICI subgroups. Subsequently, differentially expressed genes between subgroups were analyzed using the “limma” package, and genes associated with the ICI patterns were identified. Significance criteria of p. adjust < 0.05 and | Log2FC | > 1 were set to identify the significant DEGs among the different ICI subgroups.

### Dimensionality Reduction and the Construction of ICI Scores

The ICI scores were constructed following the work of Zhang et al. (2020). First, to classify the patients in the training set based on DEGs, an unsupervised clustering method was used; the positively and negatively correlated DEGs with the clustering features were called ICI gene signatures A and B, respectively. Second, the dimensionality reduction of gene signatures A and B based on ICI was performed using the Boruta algorithm, followed by subsequent extraction of the signature score (corresponding to principal component 1) using the PCA algorithm. Finally, the computation of ICI scores for each patient was according to the following equation:

$$ICI\;score={\sum PC1}_{A}-{\sum PC1}_{B}$$

### Somatic Mutations in the The Cancer Genome Atlas Cohort

The corresponding data for the patient mutations in the HCC-TCGA cohort were collated on the Mutect2 platform and were downloaded using the “TCGAbiolinks” package (Colaprico et al., 2016). The total number of nonsynonymous mutations in the samples was calculated to compare the differences in the mutation burdens between the two ICI score-based subgroups. Subsequently, using the “maftool” in R, the top 25 driver genes having the highest mutation frequency were identified and the mutation differences in the driver genes between the high- and low-score subgroups were compared (Mayakonda et al., 2018).

### Immunotherapeutic Responses of ICI Subgroup

Since different ICI subgroups may have different sensitivities to immunotherapy, the TIDE (http://tide.dfci.harvard.edu/) algorithm was used to predict the anti-PD1 and anti-CTLA4 treatment responses of patients in the TCGA and ICGC cohorts (Jiang et al., 2018b; Fu et al., 2020). Subsequently, with the aid of unsupervised subclass mapping (https://cloud.genepattern.org/gp/) (Hoshida et al., 2007), data from the high- and low-score subgroups were compared to a published dataset consisting of 47 patients’ responses to anti-PD1 and anti-CTLA4 treatments (Roh et al., 2017). This analysis predicted the immunotherapeutic responses of the high- and low-subgroups; FDR < 0.05 was set as the threshold for a significant response to anti-PD1 and anti-CTLA4 treatment. Additionally, the independent dataset IMvigor210 was used to analyze the predictive efficacy of ICI scores. The IMvigor210 dataset consisting of 298 cases of uroepithelial carcinoma samples and their corresponding clinical information, were obtained from the freely available, fully documented software and data packages under the Creative Commons 3.0 Attribution License, available at http://research-pub.gene.com/IMvigor210CoreBiologies.

### Statistical Analysis

All statistical analyses and plotting were performed using R software (version 4.04). For comparisons of more than two groups, the Kruskal-Wallis test was used, else, we used the Wilcoxon test. For the subgroups in each data set, the Kaplan-Meier plotter generated the survival curves, and the log-rank tests were determined any statistically significant differences. The correlations between ICI score for the subgroups and associated somatic mutation frequencies were evaluated and analyzed by the chi-square test. Unless stated, *p* < 0.05 (two-tailed) was considered to be statistically significant.

## Results

### Immune cell infiltrationICI Landscape in the TCGA Cohort

**FIGURE 1:**
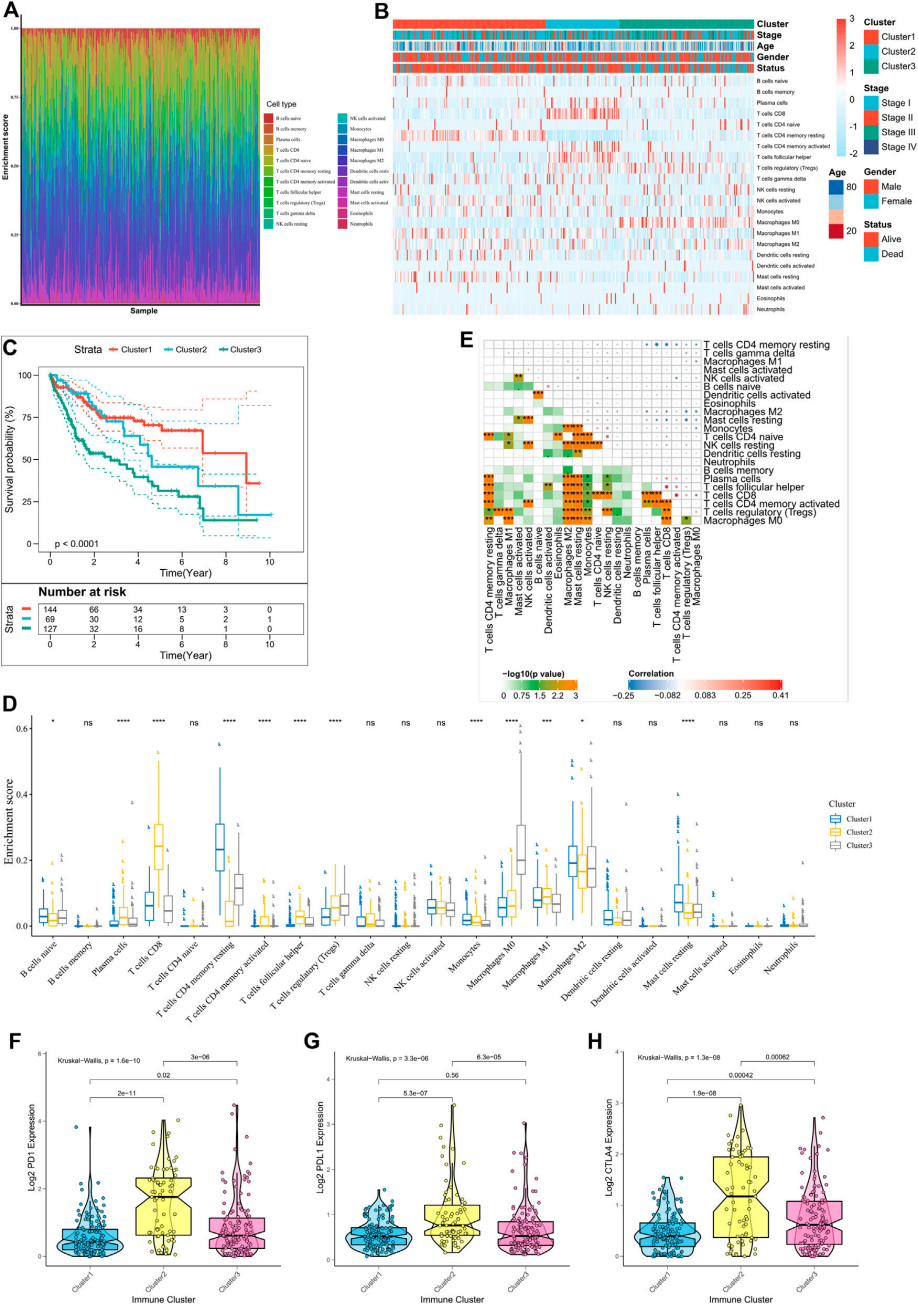
Immune landscape in the TCGA cohort. **(A)** The immune landscape of 22 ICIs in HCC patients; **(B)** Unsupervised clustering of tumor-infiltrating immune cells in the TCGA cohort, where rows represent tumor-infiltrating immune cells and columns represent samples; **(C)** Kaplan-Meier curves for overall survival (OS) of patients with different ICI clusters, where log-rank *p* = 0.018; **(D)** Proportion of tumor-infiltrating immune cells in the three ICI clusters, where Kruskal-Wallis was used to test and compare the statistical differences of the three ICI clusters. *p < 0.05; ∗∗*p* < 0.01; ∗∗∗*p* < 0.001; ∗∗∗∗*p* < 0.0001; **(E)** Cell interactions of tumor-infiltrating immune cell types. **(F–H)** Expression differences in PD-L1 **(F)**, PD1 **(G)**, and CTLA4 **(H)** between different ICI clusters (Kruskal-Wallis test, *p* < 0.0001).

Supplementary Figure S1 displayed a brief flow chart of this study. The execution of the CIBERSORT algorithm quantified the activity or enrichment of immune cells in the HCC tumor tissues (Figure 1A, Supplementary Table S1). Based on the 340 tumor samples and their corresponding ICI features in the training set, the ConsensusClusterPlus package of R software executed the unsupervised clustering method. Thus, we classified the HCC patients into three different ICI subtypes.

There were significant survival differences among the subtypes (log-rank test, *p* < 0.0001; Supplementary Figure S2A–E; Figures 1B,C); ICI cluster 1 was associated with a good prognosis while ICI cluster 3 had the worst prognosis. Additionally, to assess the intrinsic differences among the biological parameters underlying the different clinical phenotypes, ICI differences were compared between the three subgroups. ICI cluster 1 showed the highest infiltration of activated B cells, monocytes, and resting memory CD4 T cells, and the lowest infiltration of regulatory T cells. A more favorable prognosis of patients in ICI cluster 2 may be attributed to the high degree of infiltration of plasma cells, activated memory CD4T cells, M1 macrophages, and CD8T cells. However, in ICI cluster 3, higher infiltration of regulatory T cells, MO macrophages may underlie the poorest prognosis due to suppressed tumor immunity responses (Figure 1D). We also plotted the correlation heat maps to depict the interactions between immune cells in TME (Figure 1E). The expression differences in the different ICI subtypes, for three important immune checkpoints, PD1, PDL1, and CTLA4, were also analyzed. ICI cluster 2 had the highest levels of expression of immune checkpoint genes, while these were lowest in ICI cluster 1 (Figures 1F–H).

### Identification of Immunogenic Subtypes

**FIGURE 2:**
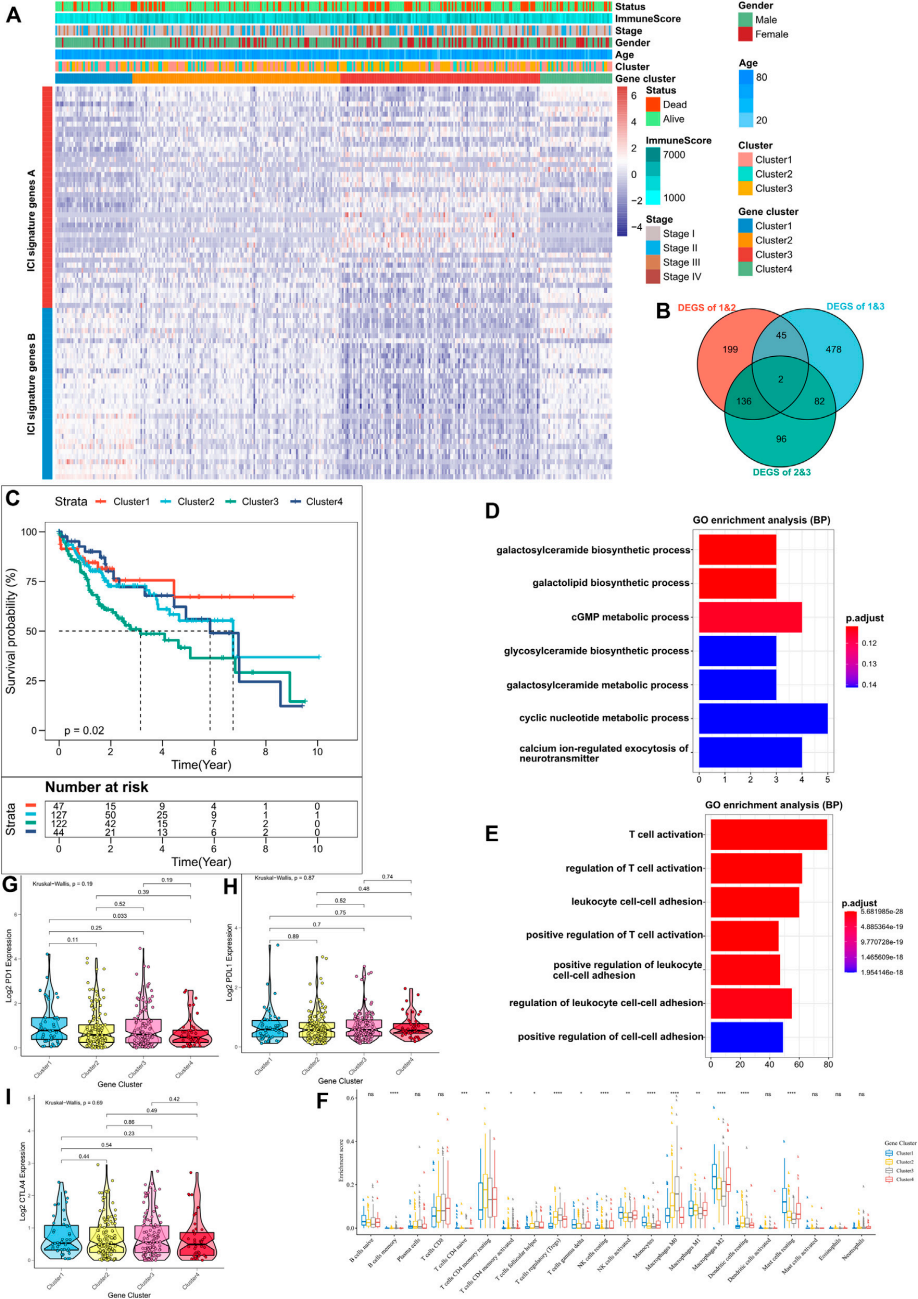
Identification of Immune-Related Gene Subtypes. **(A)** Unsupervised clustering of common DEGs in the three ICI subgroups, dividing patients into four groups; **(B)** Number of DEGs among subgroups as shown by Venn diagram; **(C)** Kaplan-Meier curves for the three subgroups of patients, where the log-rank test shows an overall *p* = 0.02; **(D,E)** Gene ontology (GO) enrichment analysis of ICI-associated signature genes: ICI signature genes A **(D)** and B **(E)**, where x-axis indicates the number of genes in each GO term; **(F)** Proportion of tumor-infiltrating immune cells in the three gene clusters, where Kruskal-Wallis was used to test and compare the statistical differences of the three ICI clusters. *p < 0.05; ∗∗*p* < 0.01; ∗∗∗*p* < 0.001; ∗∗∗∗*p* < 0.0001; **(G–I)** Expression differences of PD-L1 **(G)**, PD1 **(H)** and CTLA4 **(I)** using Kruskal-Wallis test.

To better understand the underlying biological features of different immunophenotypes, differentially expressed genes (DEGs) between these subtypes were identified using the “limma” package of the R software. A total of 1,038 DEGs were identified (Supplementary Table S2), and their intersections are shown in the Venn diagram (Figure 2B). Subsequently, based on DEGs, the ConsensusClusterPlus package was executed for unsupervised clustering analysis; thus, the TCGA cohort was divided into four gene clusters (Supplementary Figure S3A–F); Positively associated 318 DEGs in the gene clusters were defined as ICI gene feature A, and the remaining were defined as ICI gene feature B (Supplementary Table S3). Moreover, to attenuate noise and gene redundancy, dimensionality reduction of ICI gene features A and B was performed using the Boruta algorithm. The transcriptional profiles of the 78 signature DEGs identified after dimensionality reduction are shown in the heat map (Figure 2A). The significantly enhanced biological processes among the DEGs are shown in Figures 2D,E and Supplementary Table S4. Kaplan-Meier analysis showed a significant difference in survival outcomes among the four subgroups (*p* = 0.02, Figure 2C). Patients in clusters 1 and 2 had a better prognosis as compared to those in cluster 3. The presence of higher infiltration levels of M1 macrophages, monocytes, gamma delta T cell, and lower infiltration levels of regulatory T cell in clusters 1 and 2, indicated that patients in these two clusters may have a stronger anti-tumor immune response (Biswas and Mantovani, 2010; Chen and Mellman, 2017). In contrast, the highest levels of infiltration of regulatory T cell and M0 macrophage, and lowest levels of infiltration of all other immune cells in cluster 3, suggested that this may be an immune desert subtype (Biswas and Mantovani, 2010; Chen and Mellman, 2017). The concordance between the immune profiles and the prognosis using different gene clusters suggested that our classification strategy is scientifically sound and reasonably good. The levels of PD1, PDL1, and CTLA4 expression among the four clusters, however, were not significantly different (Figures 2F–H).

### Construction of the Immune Cell Infiltration Scores

**FIGURE 3:**
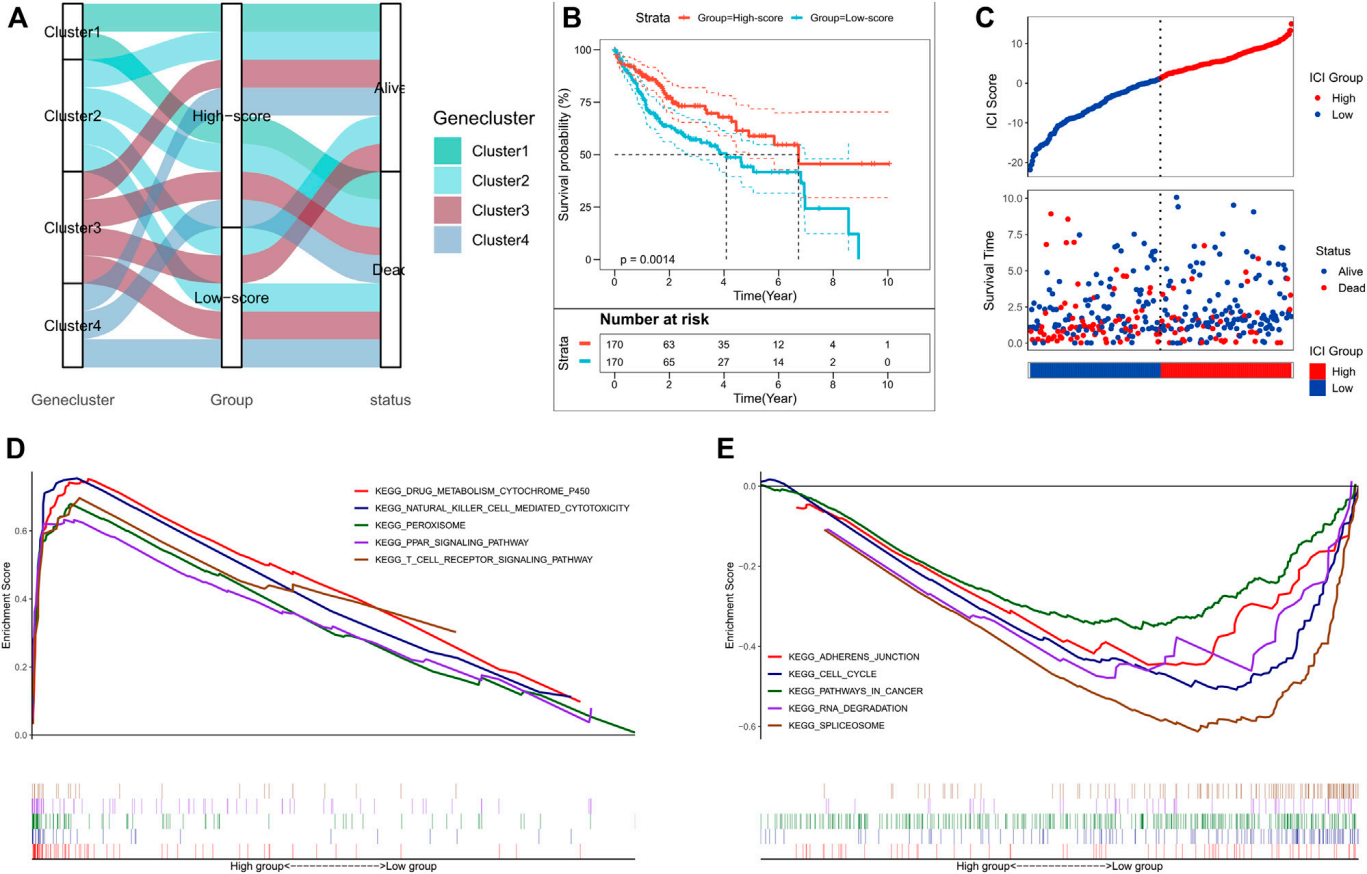
Construction of the ICI scores. **(A)** Alluvial plot of the ICI gene cluster distribution in subgroups with different ICI clusters, ICI scores, and survival outcomes; **(B)** Kaplan-Meier curves for the high and low ICI score subgroups in the TCGA cohort, where *p* = 0.0014 for the log-rank test; **(C)** Survival status of patients in the high and low ICI score subgroups in the TCGA cohort; **(D)** GSEA enrichment results for the high ICI score subgroup; **(E)** GSEA enrichment results for the low ICI score subgroup.

PCA analysis was used to quantify the ICI status of HCC patients. We calculated the sum of ICI scores A from ICI signature gene A minus the sum of ICI score B from ICI signature gene B. Thus, the prognostic feature scores defined as ICI scores were obtained. Additionally, ICI scores for the validation cohort, from ICGC were calculated using the same gene signatures A and B and the algorithm as described above. Patients were divided into high- or low-score subgroups based on median ICI score, and the distribution of patients in the four clusters is shown in Figure 3A. Kaplan-Meier analysis showed a significant difference in the prognoses between the two groups; the high-score subgroup had the best prognosis (*p* = 0.0014, Figure Figure3B). The prognostic efficacy of ICI scores was also validated in the ICGC cohort (*p* < 0.001, Supplementary Figure S4A); the high-score subgroup patients had better survival outcomes in both the TCGA and ICGC cohorts (Figure 3C; Supplementary Figure S4B). GSEA analysis showed that NK cell-mediated cytotoxicity, T cell receptor signaling, and peroxisome-related pathways were substantially enriched in the high-score subgroup, while cancer-related, cell cycle, and RNA degradation pathways were substantially enriched in the low-score subgroup (Figures 3D,E; Supplementary Table S5).

### Correlation of Immune Cell Infiltration Scores With Immune Landscape

**FIGURE 4:**
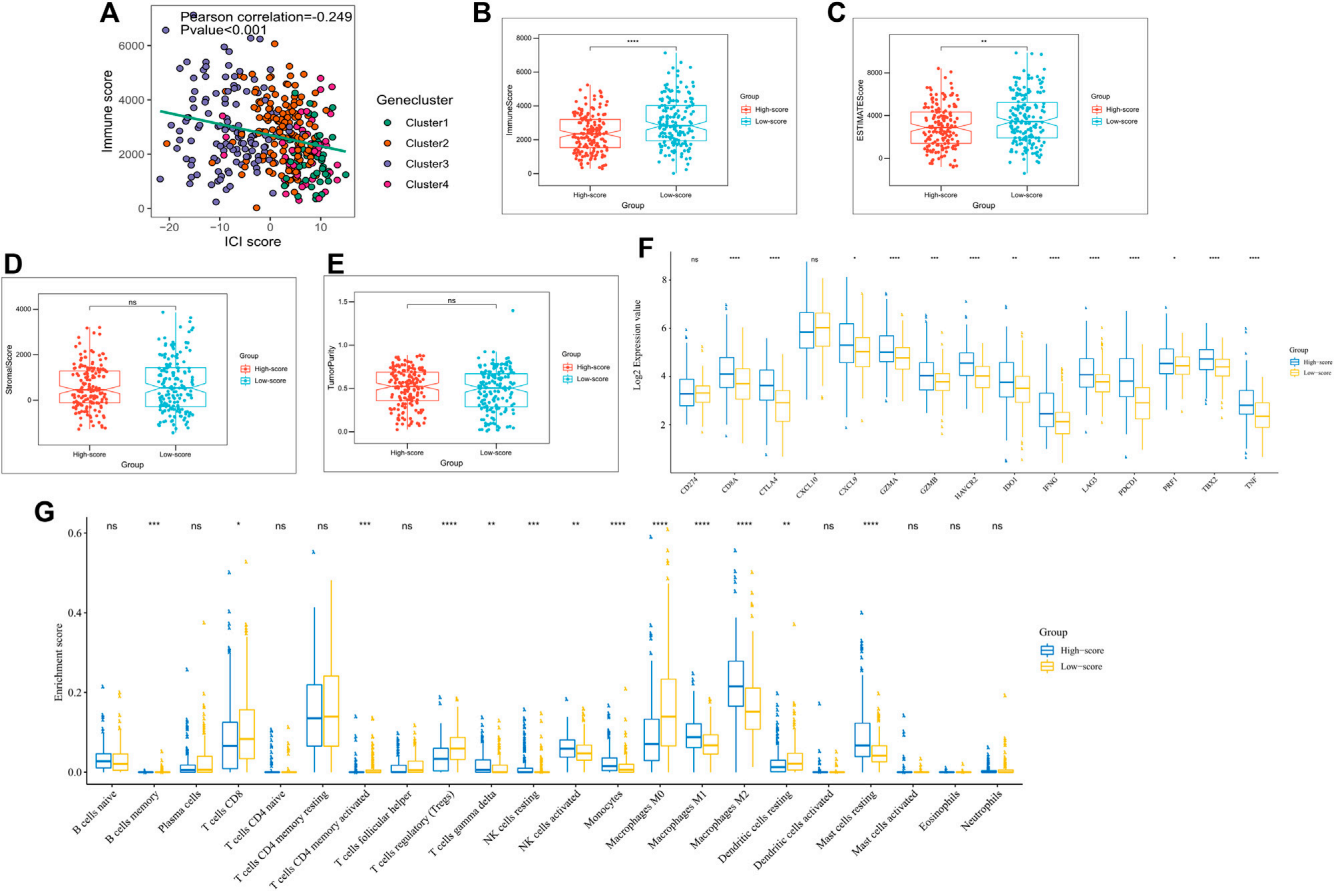
Immune landscape of subgroups with different ICI scores. **(A)** Scatter plots depicting a negative correlation between ICI scores and immune scores in the TCGA cohort, showing a Pearson correlation between ICI scores and immune scores; **(B)** Expression of immune checkpoint-related genes (IDO1, CD274, HAVCR2, PDCD1, CTLA4, and LAG3) and immunoreactive-related genes (CD8A, CXCL10, CXCL9, GZMA, GZMB, PRF1, IFNG, TBX2, and TNF) in high and low ICI score subgroups; **(C)** ICI Proportion in different ICI score subgroups; **(D)** Immune scores of the high and low ICI score groups; **(E)** ESTIMATEscores of the high and low ICI score groups; **(F)** Stromal scores in the high and low ICI score subgroups; **(G)** Tumor purity in the high and low ICI score subgroups. *p < 0.05; ∗∗*p* < 0.01; ∗∗∗∗*p* < 0.001; ∗∗∗∗*p* < 0.0001.

The immunocompetence and stromal content of the TCGA cohort were quantified using the ESTIMATE algorithm. ICI scores and immune scores were negatively correlated (Pearson correlation: R = −0.249, *p* < 0.001; Figure 4A). Box plots exhibited lower immune scores and ESTIMATE scores for the high-score subgroups (*p* < 0.05; Figures 4B,C), while stromal scores and tumor purity scores did not differ significantly between the two subgroups (Figures 4D,E). To assess immunocompetence among subgroups, CD274, CTLA4, HAVCR2, IDO1, LAG3, and PDCD1 were selected as immune checkpoint-related features, while CD8A, CXCL10, CXCL9, GZMA, GZMB, IFNG, PRF1, TBX2, and TNF were selected as immunocompetence features (Hugo et al., 2016; Ayers et al., 2017). Our results showed that almost all, immune checkpoint-related and immunocompetence-related genes (except CD274 and CXCL10), had a significant overexpression in the high ICI score subgroup (Figure 4F). Additionally, higher infiltration levels of NK cells, gamma delta T cells, monocytes, and M1 macrophage and lower infiltration levels of regulatory T cells were observed in the high-score subgroups (Figure 4G), which was also consistent in the immune landscape of the ICGC cohort (Supplementary Figures S4C,D).

### Association Between Immune Cell Infiltration Scores and Somatic Cell Variation

**FIGURE 5:**
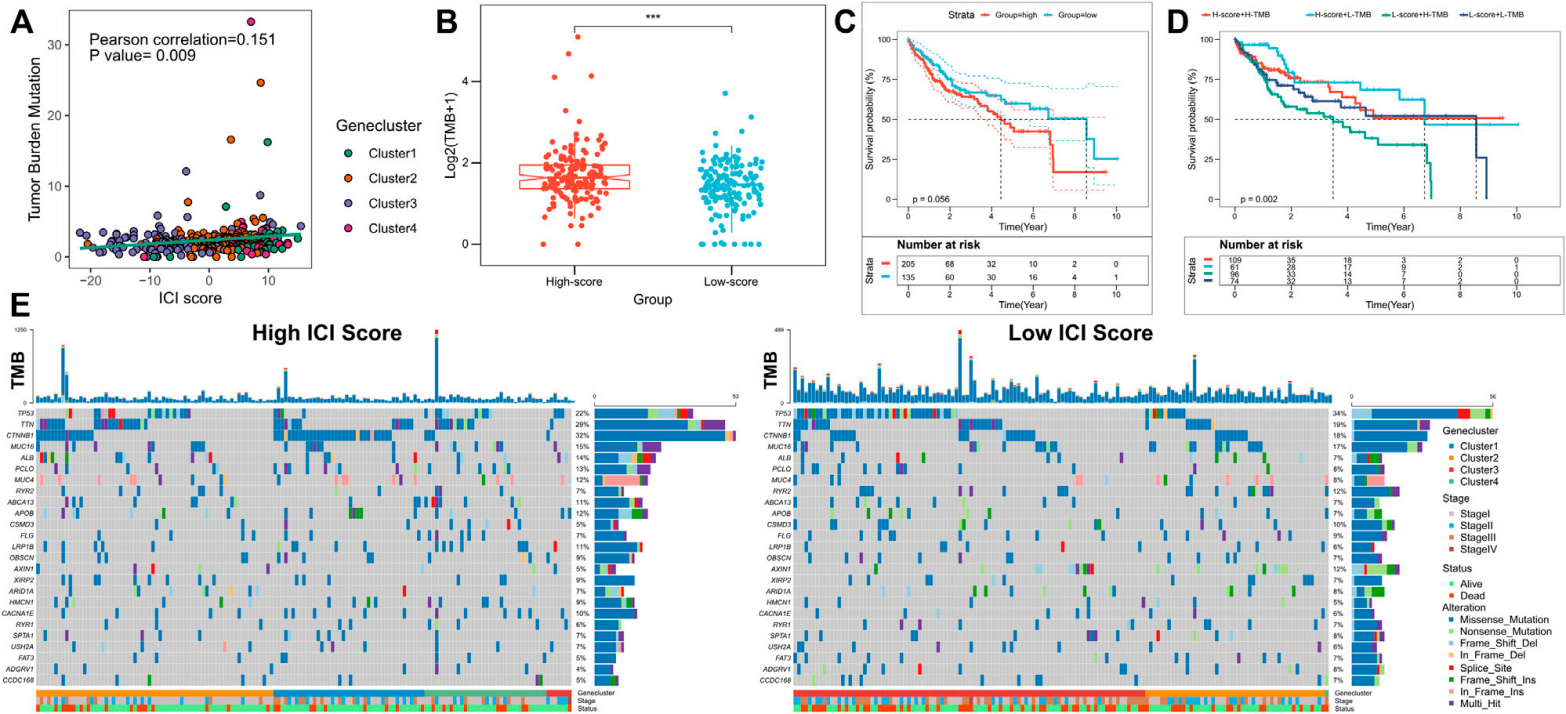
Correlation between ICI scores and somatic cell variation. **(A)** Difference in TMB between the high and low ICI score subgroups, where *p* < 0.001 for Wilcoxon test; **(B)** Scatter plot depicting a positive correlation between ICI scores and the mutational burden in the TCGA cohort, showing the Pearson correlation between ICI scores and the mutational burden; **(C)** Kaplan-Meier curves for the high and low TMB score subgroups in the TCGA cohort, where *p* = 0.056 for the log-rank test; **(D)** Kaplan-Meier curves for patients in the TCGA cohort stratified by TMB and ICI scores, where *p* = 0.002 for the log-rank test; **(E)** “oncoplot”showing the high and low ICI score subgroups for the top 25 mutant genes, with each column representing one patient.

Previous investigations have revealed that increased infiltration of CD8T cells in high mutation burden-associated tumor tissues (nonsynonymous variants) can identify and eliminate these cancers (McGranahan et al., 2016). Higher tumor mutation burden (TMB) and somatic mutation rates are associated with stronger anti-cancer immunity (Rizvi et al., 2015; Rooney et al., 2015). The KEYNOTE 012 clinical trial showed that TMB increase was associated with improved PD-1 inhibitors and prolonged progression-free survival of patients (Seiwert et al., 2016; Cristescu et al., 2018). Because of the clinical significance of TMB, the correlation between TMB and ICI scores was analyzed in detail. For this purpose, first, the TMB comparison between patients in the high- and low-score subgroups were analyzed; ICI score and TMB were positively correlated (Pearson correlation: R = 0.151, *p* = 0.009; Figure 5A). TMB was significantly higher in the high-score subgroup (Wilcoxon test *p* < 0.001; Figure 5B). Patients were divided into high- and low-TMB score subgroups based on the optimal cut-off value of TMB calculated from the “survminer” package; patients with high TMB scores exhibited poorer OS (*p* = 0.056; Figure 5C). Due to the opposing predictions of OS by ICI and TMB scores, the combined effect of these scores in the prognostic stratification of HCC was subsequently evaluated. Stratified survival analyses showed that TMB did not affect ICI score-based prediction; significant survival differences for ICI score subtypes were obtained between the two TMB-based score subgroups (log-rank test, *p* = 0.002; High TMB & High ICI score (HH) versus High TMB & Low ICI score (HL), *p* = 0.011; Low TMB & Low ICI score (LH) versus Low TMB & Low ICI score (LL), *p* = 0.047; Figure 5D). Overall, our findings suggested that ICI scores may have implications as an independent predictor of TMB and could be a reliable parameter for patient prognosis. In addition, differences in somatic variant driver genes between the low and high ICI score subgroups were evaluated. The driver genes for HCC were obtained using “maftools”; among them the most frequently altered top 25 genes were further analyzed (Figure 5E). The frequencies of CTNNB1, TP53, AXIN1, and TTN were significantly altered between the high- and low-score subgroups (chi-square test; Table 1). Taken together, these results may provide new insights for future investigations on the constituents of tumor ICI and the mechanisms of gene mutations in immune checkpoint blockade therapy.

**TABLE 1:** The association of ICI scores with somatic cell variation, where chi-square tests were used to compare statistical differences between high and low ICI score subgroups.

Gene symbol	High ICI score (%)	Low ICI score (%)	*p* Value
CTNNB1	53 (32)	30 (18)	0.0054
TP53	37 (22)	56 (34)	0.0147
AXIN1	8 (5)	19 (12)	0.0270
TTN	49 (29)	31 (19)	0.0298
ALB	23 (14)	12 (7)	0.0734
CSMD3	9 (5)	17 (10)	0.1039
LRP1B	18 (11)	9 (6)	0.1074
RYR2	11 (7)	19 (12)	0.1272
ADGRV1	7 (4)	13 (8)	0.1713
APOB	20 (12)	12 (7)	0.1934
HMCN1	15 (9)	8 (5)	0.1945
PCLO	21 (13)	13 (8)	0.2057
CACNA1E	16 (10)	9 (6)	0.2122
ABCA13	18 (11)	11 (7)	0.2441
MUC4	20 (12)	13 (8)	0.2720
FAT3	8 (5)	12 (7)	0.3635
CCDC168	9 (5)	11 (7)	0.6503
MUC16	25 (15)	28 (17)	0.6536
RYR1	10 (6)	12 (7)	0.6638
ARID1A	11 (7)	13 (8)	0.6757
SPTA1	11 (7)	13 (8)	0.6757
FLG	12 (7)	14 (9)	0.6863
OBSCN	15 (9)	12 (7)	0.6892
XIRP2	15 (9)	12 (7)	0.6892
USH2A	11 (7)	10 (6)	1.0000

### Predictive Efficacy of Immune Cell Infiltration Scores for Immunotherapy

**FIGURE 6:**
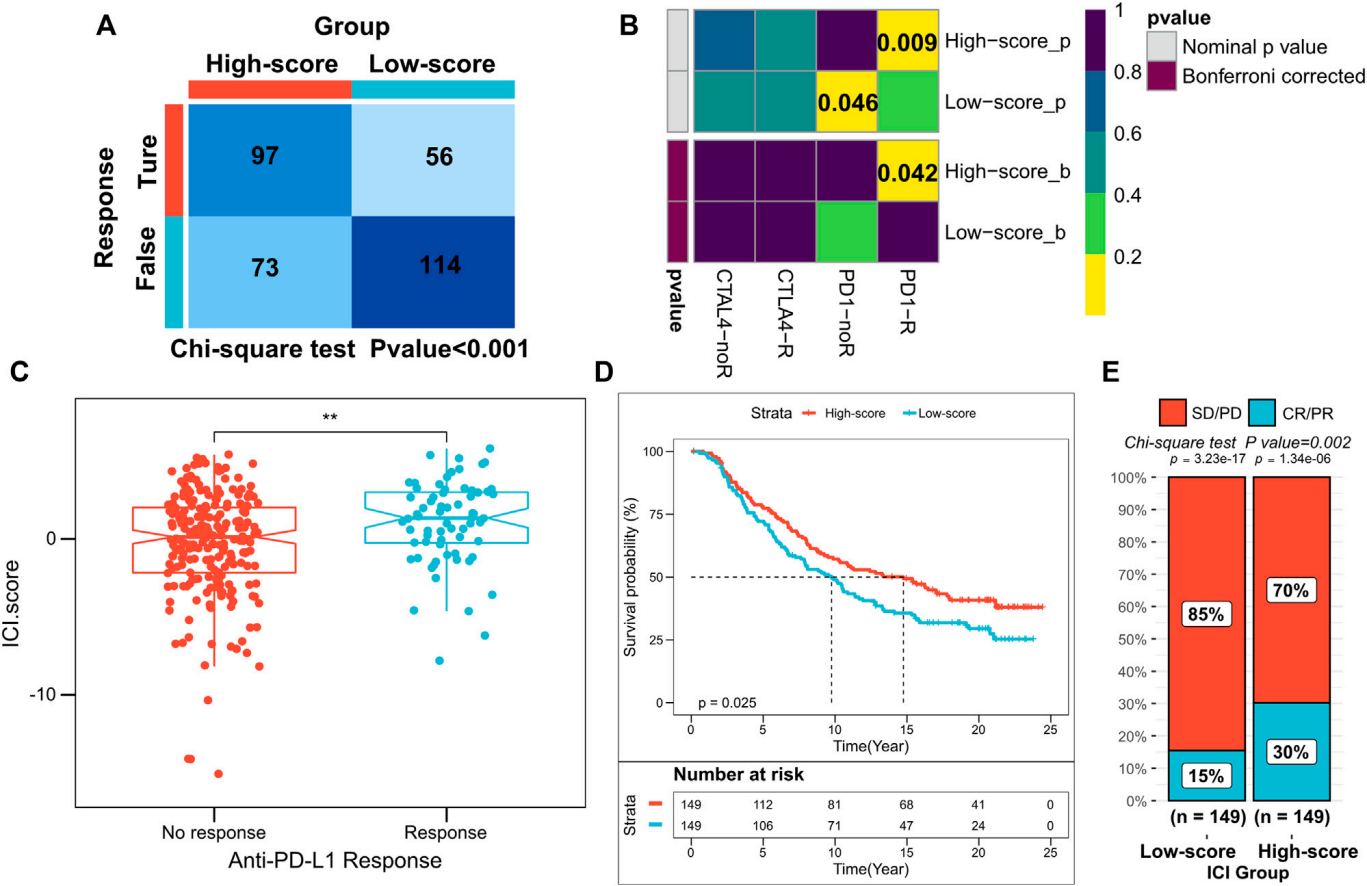
Role of ICI scores in predicting the efficacy of immunotherapy. **(A)** TIDE algorithm showing higher immunotherapy response in patients with high ICI scores, where *p* < 0.001 for the chi-square test; **(B)** Subclass mapping showing higher sensitivity to anti-PD1 treatment in patients with high-score subgroups (FDR = 0.042); **(C)** ICI scores for subgroups with different anti-PD-1 clinical response status, where *p* < 0.01 for the Wilcoxon test; **(D)** Kaplan-Meier curves for patients with high and low ICI scores in the IMvigor210 cohort, where *p* = 0.025 for the log-rank test; (d) Clinical response rates to anti-PD-L1 immunotherapy in the high or low ICI score subgroups in the IMvigor210 cohort [complete response (CR)/partial response (PR) and stable disease (SD)/progressive disease (PD)].

Novel immune checkpoint inhibition has shown promising results in both preclinical trials and real clinic settings. However, only a small proportion of patients respond to these therapies (Curran et al., 2010; Grosso and Jure-Kunkel, 2013; Larkin et al., 2015). Our subsequent analyses assessed the utility of scores based on ICI in predicting the efficacy of immunotherapy in HCC. Differences in response to anti-PD1 and anti-CTLA4 therapy between the high- and low-score subgroups of patients in the TCGA and ICGC cohorts were evaluated using the TIDE algorithm. In the high-score subgroup, the patients had a higher immunotherapy response rate (chi-square test *p* < 0.001; Figure 6A; Supplementary Figure S4E). Subclass mapping analysis predicted the immunotherapy responses of both subgroups to PD1 and CTLA4 inhibitors. The high-score subgroups in both the TCGA and ICGC cohorts were found to be more sensitive to anti-PD1 treatment (TCGA: FDR = 0.042; ICGC: FDR = 0.022, respectively) (Figure 6B; Supplementary Figure S4F). In addition, patients in the IMvigor210 cohort administered with anti-PD-L1 immunotherapy were also assigned the corresponding ICI scores (high or low). Notably, patients with high ICI scores in the IMvigor210 cohort survived longer as compared to those with low ICI scores (log-rank test, *p* = 0.0017; Figure 6C). In the IMvigor210 cohort, anti-PD-L1 therapy’s objective remission rate was significantly higher in the high ICI score subgroup (Chi-square test, *p* = 0.002; Figure 6D). Our results also indicated that higher ICI scores in the IMvigor210 cohort were associated with objective responses to anti-PD-L1 therapy (Wilcoxon test, *p* < 0.01; Figure 6E). Overall, these findings suggest a possible association between ICI scores and immunotherapeutic responses.

## Discussion

HCC is an aggressive tumor with a high degree of malignancy, and most patients are diagnosed initially at an advanced stage (Zhou et al., 2020). High recurrence and metastasis rates of advanced HCC to a low possibility for surgical resection (Zhou et al., 2020; Feng et al., 2021). Within the local area, the complex genomic alterations, differences in biological behaviors, and heterogeneity of the tumor microenvironment resulting in a complex HCC process. Currently, immunotherapy is a promising treatment strategy available for HCC (Huang et al., 2020). Due to the limitations of surgical resection, chemotherapy and immunotherapy have received increasing attention in the treatment of advanced HCC (Brown et al., 2019). However, immunotherapeutic response rates are highly heterogeneous and remain considerably low (Feng et al., 2021). Thus, in HCC, immune-related classification criteria may provide new insights to assess the efficacy of immunotherapy and predict the patient prognosis.

The high genomic heterogeneity of HCC results in the complexity of the immune microenvironment (Dal Bo et al., 2020). Therefore, the identification of novel signatures in HCC based on immune-related genes provides a new direction for assessing the efficacy of immunotherapy. Further assessment of these classifications based on gene signatures may help in developing immunotherapy strategies with improved sensitivity for different subtypes of HCC. Zhang et al. characterized the ICI dynamics in HCC by single-cell sequencing, and thus provided a new basis for investigations of the immune landscape (Zhang et al., 2019a). Sia et al. identified active or depleted immune subtypes in HCC based on immune gene transcriptional profiling. This suggests that active immune subtypes may be more sensitive to immunosuppressant therapy (Sia et al., 2017). Zhang et al. integrated multi-omics data and show new immunophenotypic classifications in HCC which may be useful for prognostic prediction and potentially supporting new treatment targets (Zhang et al., 2019b). Indeed, these studies have their unique strengths and potential and complement each other. Therefore, investigations of HCC immune subtypes from different perspectives hold great promise for research, and a better classification of immune features would enhance the overall understanding of HCC immunotherapy.

In the present study, we analyzed the classical HCC dataset from the TCGA and ICGC cohorts and divided the patients into three different immune subtypes. Our results suggested that high infiltration levels of CD4 T cells, CD8 T cells, and M1 macrophage and low infiltration levels of regulatory T cells were associated with good prognosis, consistent with previous studies (Rooney et al., 2015; He et al., 2018). Due to the heterogeneity of immune landscape and prognosis among the three immune subtypes, we speculated that an integrated ICI profile analysis and evaluation of immune-based gene expression patterns would be a new approach to develop patient-customized and tailored treatment strategies. Four distinct gene clusters were obtained based on differentially expressed genes between the subtypes; clusters 1 and 2 exhibited a more favorable immune activation phenotype, exhibited higher infiltration of M1 macrophages, monocytes, gamma delta T cells, and lower infiltration levels of regulatory T cells (Biswas and Mantovani, 2010; Chen and Mellman, 2017); in contrast, the highest infiltration levels of regulatory T-cells and M0 macrophage and lowest infiltration levels of other cell types were found in cluster 3, which suggested a general immune failure phenotype (Biswas and Mantovani, 2010; Chen and Mellman, 2017). TME impact on patient’s OS has been well documented in previous studies; ICI differences resulted in cluster 1 and cluster 2 patients having a good prognosis, while patients in cluster 3 had the worst prognosis, consistent with previous studies (Chen et al., 2019; Li et al., 2019). These findings suggested that the gene clusters in this study may have a potential role in more accurate predictions of patient outcomes.

Given the differences in patient prognosis and immune landscape between gene clusters, it was imperative to quantify the individual patient ICI patterns for improved outcome prediction. Individual models based on tumor subtype-specific biomarkers show good efficacy for HCC (Sia et al., 2017; Kurebayashi et al., 2018). In this study, potential “subtype biomarkers” were obtained using the Boruta algorithm and ICI scores were calculated to quantify ICI patterns. GSEA showed that cancer-related pathways including cell cycle pathways and RNA degradation pathways were significantly enriched in the low ICI score group. Recently, preclinical trial reports show the correlation of gene mutations with tolerance or immunotherapeutic responses (Rizvi et al., 2015; Rooney et al., 2015). Several genes with significant differences in mutation frequencies exist between the high and low ICI score subgroups. All of these play an important role in cancer progression (Mazzoni and Fearon, 2014; Mantovani et al., 2019; Wen et al., 2019; Yang et al., 2020). Moreover, the highly immunotherapy-sensitive, TMB, was significantly lowered in patients with lower ICI scores (correlation 0.151). The stratified analysis could independently predict the prognostic value of ICI scores for TMB. These results implied that ICI scores and TMB represented different aspects of tumor immunobiology and ICI scores could indeed predict patient responses to immunotherapy in conditions independent of TMB.

The efficacy of ICI scores in predicting immunotherapeutic responses was further evaluated by multiple methods; TIDE and subclass mapping analyses showed that patients with higher ICI scores were more sensitive to anti-PD1 therapy. After evaluating patients in the anti-PD1 immunotherapy regime in the IMvigor210 cohort, a significant increase in ICI scores was found which validated the predictive value of patients’ response. These results indicated that mono-immunotherapy may benefit patients with high ICI scores.

However, the present study has some limitations. The current results need to be validated for their efficiency in immunotherapy clinical trials with larger HCC cohorts. This would confirm the utility of classification for clinical evaluation and decision-making. Additionally, transcriptomic information was obtained from post-surgical liver tissues. Thus, the model may not accurately predict outcomes prior to the onset of HCC. Therefore, a better understanding of circulating biomarkers released into the bloodstream from tumor cells and tumor-associated immune cells is important. Further *in vivo* and *in vitro* experiments should investigate the potential functional and mechanical differences between the subtypes. Finally, the findings of this study and ICI scores may apply to other cancers, and these require further studies.

In conclusion, a comprehensive analysis of ICI patterns in HCC provides a foundational basis for the regulation of anti-tumor/tumor-promoting immune responses in HCC. These suggested that differences in ICI patterns correlated with tumor heterogeneity and therapeutic complexity. Based on this, a practical model for quantifying individual ICI patterns was proposed, which could predict the prognosis of HCC patients and identify potential candidates for developing immunotherapy regimens.

## Data Availability

The original contributions presented in the study are included in the article/Supplementary Material, further inquiries can be directed to the corresponding author.

## Abbreviations

CDF: Cumulative distribution function; CR: Complete response; DEGs: differentially expressed genes; FDR: false discovery rate; FKPM: Fragments Per Kilobase per Million; GO: Gene Ontology; GSEA: Gene set enrichment analysis; HCC: Hepatocellular carcinoma; ICGC: International Cancer Genome Consortium; ICI: Immune Cell Infiltration; KEGG: Kyoto Encyclopedia of Genes and Genomes; PD: Progressive disease; PR: Partial response; SD: Static disease; TME: Tumor Microenvironment; TCGA: The Cancer Genome Atlas; TPM: Transcripts Per Million; TMB: Tumor Mutation Burden; TIDE: Tumor Immune Dysfunction and Exclusion.
